# Different Genetic Sources Contribute to the Small RNA Population in the Arbuscular Mycorrhizal Fungus *Gigaspora margarita*

**DOI:** 10.3389/fmicb.2020.00395

**Published:** 2020-03-13

**Authors:** Alessandro Silvestri, Massimo Turina, Valentina Fiorilli, Laura Miozzi, Francesco Venice, Paola Bonfante, Luisa Lanfranco

**Affiliations:** ^1^Department of Life Sciences and Systems Biology, School of Nature Sciences, University of Turin, Turin, Italy; ^2^Institute for Sustainable Plant Protection, Italian National Research Council, Turin, Italy

**Keywords:** *Gigaspora margarita*, arbuscular mycorrhizal fungi, small RNA, RNA interference, viruses, symbiosis

## Abstract

RNA interference (RNAi) is a key regulatory pathway of gene expression in almost all eukaryotes. This mechanism relies on short non-coding RNA molecules (sRNAs) to recognize in a sequence-specific manner DNA or RNA targets leading to transcriptional or post-transcriptional gene silencing. To date, the fundamental role of sRNAs in the regulation of development, stress responses, defense against viruses and mobile elements, and cross-kingdom interactions has been extensively studied in a number of biological systems. However, the knowledge of the “RNAi world” in arbuscular mycorrhizal fungi (AMF) is still limited. AMF are obligate mutualistic endosymbionts of plants, able to provide several benefits to their partners, from improved mineral nutrition to stress tolerance. Here we described the RNAi-related genes of the AMF *Gigaspora margarita* and characterized, through sRNA sequencing, its complex small RNAome, considering the possible genetic sources and targets of the sRNAs. *G. margarita* indeed is a mosaic of different genomes since it hosts endobacteria, RNA viruses, and non-integrated DNA fragments corresponding to mitovirus sequences. Our findings show that *G. margarita* is equipped with a complete set of RNAi-related genes characterized by the expansion of the *Argonaute-like* (*AGO-like*) gene family that seems a common trait of AMF. With regards to sRNAs, we detected populations of sRNA reads mapping to nuclear, mitochondrial, and viral genomes that share similar features (25-nt long and 5′-end uracil read enrichments), and that clearly differ from sRNAs of endobacterial origin. Furthermore, the annotation of nuclear loci producing sRNAs suggests the occurrence of different sRNA-generating processes. *In silico* analyses indicate that the most abundant *G. margarita* sRNAs, including those of viral origin, could target transcripts in the host plant, through a hypothetical cross-kingdom RNAi.

## Introduction

RNA interference (RNAi) or RNA silencing is a conserved eukaryotic pathway involved in the repression of gene expression at transcriptional or post-transcriptional level (Moazed, 2009; Wilson and Doudna, 2013; Ipsaro and Joshua-Tor, 2015). RNAi carries out several biological functions, such as gene regulation, defense against mobile repetitive DNA sequences, retroelements, transposons, and viruses. The process is mediated by small RNAs (sRNAs) of about 20–30 nucleotides that direct, by sequence complementarity, the recognition and silencing of the target genetic elements. The RNAi pathway relies on three core enzymes: Dicer-like (DCL), Argonaute-like (AGO-like), and RNA-dependent RNA polymerase (RdRp). DCL are ribonuclease III (RNase III) proteins that cleave double-stranded RNAs (dsRNAs) or single-stranded hairpin RNAs producing sRNAs that are then loaded onto AGO-like which, guided by sRNAs, are responsible for the silencing of the specific target sequences. RdRp generally play a dual role, both triggering the RNAi pathway and/or amplifying the silencing signals through the synthesis of dsRNAs from aberrant RNAs.

In 2013, Weiberg et al. (2013) discovered that RNAi is also a key molecular component of interspecies communication: sRNAs can be transferred across the contact interface of two interacting organisms and, acting as pathogen effectors, they silence specific genes in host cells in order to favor colonization. This phenomenon, known as cross-kingdom RNAi, occurs in several pathogenic and parasitic interactions (Weiberg et al., 2013; Mayoral et al., 2014; Zhang et al., 2016; Shahid et al., 2018; Chow et al., 2019; Cui et al., 2019) where it can function as an attack or a defense strategy. Interestingly, it was also recently described in the legume-rhizobium symbiosis where bacterial transfer RNA (tRNA)-derived small RNA fragments are signal molecules that modulate host gene expression and nodule formation (Ren et al., 2019). It has been proposed that sRNAs can also be exchanged between the partners of the arbuscular mycorrhizal (AM) symbiosis (Huang et al., 2019), a very ancient mutualistic association established between the roots of most plants and the obligate biotrophic fungi belonging to Glomeromycotina (Mucoromycota phylum; Spatafora et al., 2016), known as arbuscular mycorrhizal fungi (AMF) (Lanfranco et al., 2018). Indeed, host-induced (HIGS) and virus-induced gene silencing (VIGS) have been successfully employed to silence AMF genes expressed during root colonization (Helber et al., 2011; Kikuchi et al., 2016; Tsuzuki et al., 2016; Xie et al., 2016; Voß et al., 2018).

The RNAi machinery and the sRNA populations in AMF have been characterized only in the model AMF species *Rhizophagus irregularis* (Lee et al., 2018; Silvestri et al., 2019). The availability of the full genome sequence of *Gigaspora margarita* (Venice et al., 2019) offered the possibility to explore how conserved are RNAi features in AMF. *G. margarita* is of particular interest as it belongs to Gigasporaceae, an early diverging AMF group, well separated from Glomeraceae that includes *R. irregularis* (Krüger et al., 2012). It has a complex genomic structure with the largest fungal genome so far annotated (773 Mbp) and a rich content (64%) in transposable elements. In addition, the isolate used for the genome project (BEG34) can be treated as a meta-organism, since it hosts the obligate endobacterium *Candidatus* Glomeribacter gigasporarum (CaGg; Ghignone et al., 2012) and six viral species (Turina et al., 2018), whose genome sequences are available. Notably, for the four mitoviruses present in *G. margarita* we could also prove the existence of DNA fragments corresponding to portions of their genome: this feature, never found in mycoviruses, has been described as an anti-viral response in insects (Goic et al., 2016).

In this work we described the RNAi-related gene components in *G. margarita* and the sRNA population originating from its metagenome. *G. margarita*, in analogy to *R. irregularis*, is equipped with a complete set of RNAi-related genes, characterized by the expansion of the *AGO*-like family, as well as sRNAs. A population of nuclear DNA mapping sRNAs substantially different from that of *R. irregularis* was found. The high level of sRNA reads mapping to viral genomes suggests that *G. margarita* RNAi machinery is able to provide an antiviral defense. Furthermore, through an *in silico* analysis, we identified a group of plant genes that can be potentially targeted by the most expressed *G. margarita* sRNAs.

## Materials and Methods

### Biological Material

The spores of *G. margarita* strain BEG34 were obtained from *Trifolium repens* plants inoculated with 100–150 spores. After 3 months of growth with night/day temperature conditions of 21 (night) and 23°C (day), new spores were collected by wet sieving technique, divided in batches of 100 and vernalized in distilled water for a week in the dark at 4°C. Spores were then surface-sterilized with chloramine T (3% W/V) and streptomycin sulfate (0.03% W/V), washed with sterile distilled water and incubated in 1 ml of sterile distilled water for a week in the dark at 30°C to allow germination. Finally, germinated spores were collected, immediately frozen in liquid nitrogen, lyophilized and stored at −80°C.

### RNA Extraction for sRNA-Seq

Batches of 100 germinated spores were ground in a bead beater with 3-mm tungsten beads at 18 Hz/s for 3 min. Total RNA was extracted with Direct-zol^TM^ RNA MiniPrep (Zymo Research) kit, performing the in column DNase I treatment as recommended by the manufacturer. RNA concentration and quality were assessed with a Nanodrop1000 (Thermo Scientific). Three biological replicates (SG1, SG2, and SG3) were prepared pooling together, for each replicate, same amount of RNA extracted from two independent biological samples (so a total of six biological samples were used). Samples were then delivered to Macrogen (South Korea) for RNA integrity check, library preparations, and sequencing.

### Identification and Phylogenetic Analyses of RdRp, DCL, and AGO-Like

Screening the Pfam annotation of the *G. margarita* proteome (Venice et al., 2019), we retrieved all the sequences containing a “Piwi” (Pfam ID: PF02171.17), an “RdRP” (Pfam ID: PF05183.12), or two “Ribonuclease_3” (Pfam ID: PF00636.26) domains, and we considered them as AGO-like, RdRp, or DCL, respectively. The whole amino acid sequences of DCL, AGO-like, and RdRp were aligned with MAFFT v7.310 (option: –auto) (Katoh and Standley, 2013) together with fungal sequences analyzed in Silvestri et al. (2019). Their phylogenetic relationships were inferred by the Maximum-Likelihood method implemented in the IQ-TREE software (options: -m TEST -bb 1000 -alrt 1000 -o “root”) (Nguyen et al., 2015). The software performed model selection (Kalyaanamoorthy et al., 2017), tree reconstruction, and branch support analysis by ultra-fast bootstrap method (1000 replicates) (Hoang et al., 2018). Trees were reshaped on root with Newick Utilities v1.6 (command: nw_reroot) (Junier and Zdobnov, 2010) and visualized with Evolview v3 (Subramanian et al., 2019).

### Bioinformatics Pipeline

Raw sRNA-seq reads were checked for quality with FastQC (Babraham Bioinformatics) and then cleaned from adapters (TGGAATTCTCGGGTGCCAAGG), artifacts (default parameters), and low quality reads (-q 28 -p 50) with Fastx Toolkit (Hannon Lab). Further filtering of raw reads was performed with Bowtie (Langmead et al., 2009), by removing the reads that mapped with up to 1 mismatch to the tRNA, rRNA, snRNA, and snoRNA sequences from Rfam 12.0 database (Nawrocki et al., 2015), and those mapping with 0 mismatches to all the “ribosomal RNA” sequences present in GenBank for Mucoromycota taxonomy; we finally kept only the 18- to 35-nt long reads. Filtered reads were mapped with no mismatch with bowtie to the unmasked versions of *G. margarita* nuclear (Venice et al., 2019), mitochondrial (Pelin et al., 2012), *Ca*Gg (Ghignone et al., 2012), and viral (Turina et al., 2018) genomes. A set of Bash, Perl, and R scripts were used for the analysis and visualization of nucleotide length distribution, 5′-end nucleotide composition, and reads redundancy. For the analysis of nucleotide length distribution of sRNAs in *R. irregularis*, reads from “extra-radical mycelium” libraries of a previous study (Silvestri et al., 2019) were mapped to mitochondrial (NCBI accession: JQ514224.2) and nuclear genomes (Chen et al., 2018) of *R. irregularis* with no mismatch using bowtie.

ShortStack v.3.8.5 (Johnson et al., 2016) was used for genome-guided sRNA-generating loci prediction and annotation on *G. margarita* nuclear genome (options: –mismatches 0 –foldsize 1000 –dicermin 18 –dicermax 35 –pad 200 –mincov 10.0rpmm). BEDTools (Quinlan and Hall, 2010) was used to compare the genomic locations of sRNA-generating loci with those of annotated protein-encoding genes and of annotated transposable elements (Venice et al., 2019). PCA on sRNA-generating loci was performed in R with “FactoMineR” v1.42 (Lê et al., 2008) and “factoextra” v1.0.5^1^ packages. HDBSCAN clustering was performed (parameters: minPts = 20) with dbscan R package v1.1-4 (Hahsler et al., 2019). Homology analysis of *G. margarita*-(*Gma)*-sRNA-generating loci with fungal repetitive elements from RepBase 23.04^2^ was performed with tblastx (*E*-value ≤ 0.00005) (Camacho et al., 2009). The miRNA-like locus was annotated by ShortStack v. 3.8.5 (Johnson et al., 2016) and its secondary structure was predicted and visualized with StrucVis v.0.3^3^.

For target prediction analysis, we selected the 21-nt long sRNAs with expression level greater than 100 RPM (“Reads Per Million mapped reads”; considering only the 21–24-nt long reads mapped to *G. margarita* genome) and the 21-nt viral sRNAs with expression level >100 RPM among all viral sRNAs. These sRNA reads were then used to predict targets in *M. truncatula* A17 transcriptome (v.4.0 cDNAs on EnsemblPlants database; Zerbino et al., 2018) through psRNAtarget (2017 update; Dai et al., 2018) with default parameters and we kept only the predictions with expectation <3. GO enrichment analysis of target transcripts was performed with AgriGO (*p*-value < 0.01; statistical test: Fisher’s test with Yekutieli correction; Tian et al., 2017), using Plant Go Slim ontology. Similarly, two further target prediction analyses on *M. truncatula* transcriptome were performed as described above using only the most abundant 21-nt long sRNAs of the AMF *R. irregularis* (>100 RPM, considering only the 21–24-nt long reads mapping on the genome) from “mycorrhizal roots” libraries described in Silvestri et al. (2019) and the most abundant 21-nt long sRNAs of the non-AMF *Aspergillus fumigatus* (>100 RPM before genome mapping; Özkan et al., 2017). To identify the *A. fumigatus* sRNA sequences, two already published sRNA-seq libraries (SRA ID: SRR1583955, SRR1583956; Özkan et al., 2017) were cleaned from adapters (AGATCGGAAGAGCACACGTCT), artifacts, low qualities reads and tRNA-, rRNA-, snRNA-, and snoRNA-related sequences as described above. The remaining reads were mapped with no mismatch with bowtie to the unmasked version of *A. fumigatus* Af293 genome from Ensembl database (ASM265v1). We kept, for the target prediction on the *M. truncatula* transcriptome, only the 21-nt long genome mapped sRNAs with an abundance greater than 100 “reads per million reads” of the whole filtered sRNA libraries.

## Results and Discussion

### The Comparative Analysis of Fungal RNAi-Related Proteins Reveals Common AMF Traits

Recent surveys of two AMF genomes allowed a first characterization of the RNAi components (*DCL*, *AGO-like*, and *RdRp*) in this group of obligate biotrophs (Lee et al., 2018; Silvestri et al., 2019). These works highlighted that the two analyzed AMF, *R. irregularis* and *R. clarus*, are equipped with a RNAi machinery characterized by 26–40 AGO-like, 3–21 RdRp, and 1–2 DCL proteins. Here we analyzed the genome of the AMF *G. margarita* (Venice et al., 2019) in order to define the conservation level of the RNAi-related genes in the Glomeromycotina subphylum. Keeping only one virtual transcript for each gene, we obtained a total of 11 AGO-like, 6 RdRp, and 1 DCL corresponding proteins.

**FIGURE 1:**
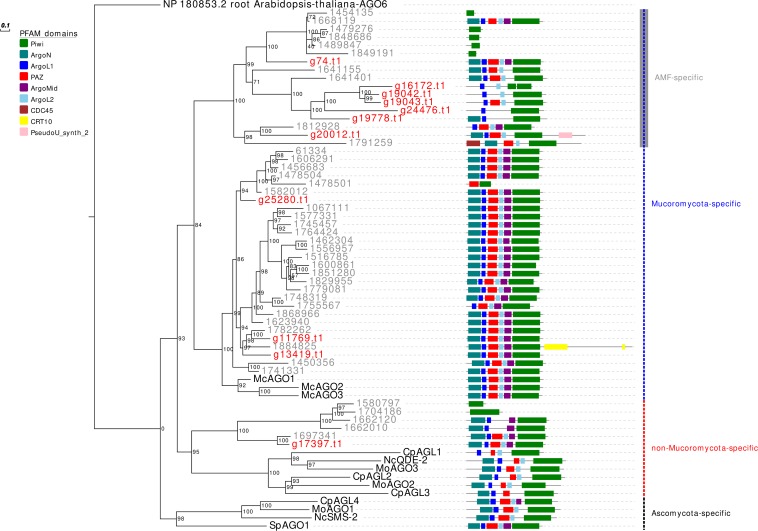
Phylogenetic analysis and PFAM domains of AGO-like proteins. Sequences are discernible by species according to a two-letter prefix and/or a color code: Mo, *Magnaporthe oryzae*; Nc, *Neurospora crassa*; Mc, *Mucor circinelloides*; Sp, *Schizosaccharomyces pombe*; Cp, *Cryphonectria parasitica*; gray, *Rhizophagus irregularis*; red, *Gigaspora margarita*. Protein ID (NCBI or JGI): MoAGO1 = XP_003716704.1, MoAGO2 = XP_003717504.1, MoAGO3 = XP_003714217.1, NcQDE-2 = XP_011394903.1, NcSMS-2 = EAA29350.1, SpAGO1 = O74957.1, McAGO-1 = 104,161, McAGO-2 = 195,366, McAGO-3 = 104,163, CpAGL1 = ACY36939.1, CpAGL2 = ACY36940.1, CpAGL3 = ACY36941.1, CpAGL4 = ACY36942.1. *R. irregularis* proteins are identified by JGI numeric codes. *G. margarita* proteins are identified by Venice et al. (2019) annotation code. The numbers at the nodes are bootstrap values (%) for 1000 replications. Tree was rooted using *Arabidopsis thaliana* Argonaute 6 (NCBI Reference Sequence: NP_180853.2). Tree was reshaped on root with Newick Utilities v1.6 and visualized with Evolview v3.

In analogy to the other analyzed AMF, *R. irregularis*, and *R. clarus*, *G. margarita* is characterized by an expansion, even if less pronounced compared to both *Rhizophagus* species, of the *AGO-like* gene family. Other filamentous fungi in the Ascomycota and Basidiomycota, in fact, typically possess 1–4 *AGO* (Chang et al., 2012). Only 5 out of the 11 *G. margarita* AGO-like proteins (g74.t1, g25280.t1, g11769.t1, g13419.t1, and g17397.t1) show all the typical AGO domains (piwi, PAZ, MID, and N-terminal) (Poulsen et al., 2013); the remaining 6 (g16172.t1, g19042.t1, g19043.t1, g24476.t1, g19778.t1, and g20012.t1) lack at least one of the non-piwi domains (Figure 1). We can speculate that those atypical AGO-like can play different biological functions, unrelated to the classic RNAi pathway. A phylogenetic analysis of fungal AGO-like revealed that 10 out of 11 *G. margarita* proteins (g74.t1, g16172.t1, g19042.t1, g19043.t1, g24476.t1, g19778.t1, g20012.t1, g25280.t1, g11769.t1, and g13419.t1) belong to a well-supported clade containing only proteins from Mucoromycota species (*G. margarita*, *Mucor circinelloides*, and *R. irregularis*; Mucoromycota-specific clade) while the remaining one (g17397.t1) groups with some AGO from Ascomycota (*Cryphonectria parasitica*, *Neurospora crassa*, and *Magnaporthe oryzae*), and 5 *R. irregularis* AGO-like (non-Mucoromycota-specific clade; Figure 1). Interestingly, the Mucoromycota-specific clade can be further divided in two subgroups, one that is AMF specific (*G. margarita* and *R. irregularis*). A third group, non-related to the previous two, contains only some Ascomycota AGO-like proteins (*C. parasitica*, *M. oryzae*, *N. crassa*, and *Schizosaccharomyces pombe*; Ascomycota-specific clade). Moreover, *G. margarita* is not equipped with small peptide-encoding ORF containing only the piwi domain that were found in *R. irregularis* but not in *R. clarus* (Silvestri et al., 2019). Despite the different number of AGO-like, *R. irregularis* and *G. margarita* are equipped with the same core set of homologous sequences: *G. margarita* possesses at least one AGO-like protein for each phylogenetic subgroup present in *R. irregularis*.

With regards to the RdRp, we found six proteins in *G. margarita*, more than the one to five generally possessed by non-AMF (Chang et al., 2012; Chen et al., 2015). In our previous work we reported 21 RdRp in *R. irregularis*, although Lee et al. (2018), using more stringent annotation criteria, only reported three.

**FIGURE 2:**
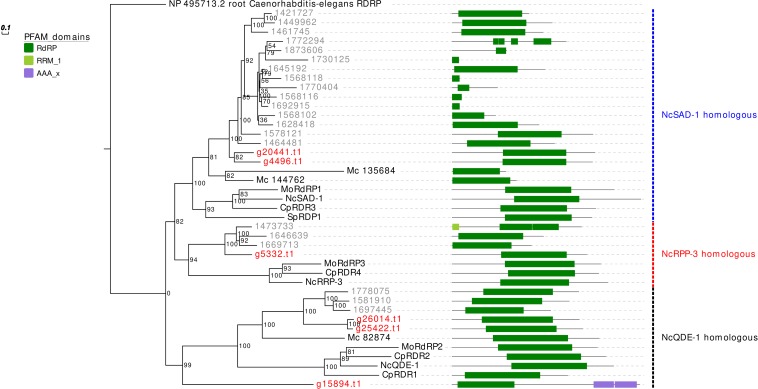
Phylogenetic analysis and PFAM domains of RdRp proteins. Sequences are discernible by species according to a two-letter prefix and/or a color code: Mo, *Magnaporthe oryzae*; Nc, *Neurospora crassa*, Mc, *Mucor circinelloides*; Sp, *Schizosaccharomyces pombe*; Cp, *Cryphonectria parasitica*; gray, *Rhizophagus irregularis*; red, *Gigaspora margarita.* Protein ID (NCBI or JGI): MoRdRP1 = XP_003721007.1, MoRdRP2 = XP_003711624.1, MoRdRP3 = XP_003712093.1, NcQDE-1 = EAA29811.1, NcSAD-1 = XP_964248.3, NcRRP-3 = XP_963405.1, SpRDP1 = NP_001342838.1, McRdRP-1 = 111871, McRdRP-2 = 104159, CpRDR1 = 270014, CpRDR2 = 35624, CpRDR3 = 10929, CpRDR4 = 339656. *R. irregularis* proteins are identified by JGI numeric codes. *G. margarita* proteins are identified by Venice et al. (2019) annotation code. The numbers at the nodes are bootstrap values (%) for 1000 replications. Tree was rooted using *Caenorhabditis elegans* RdRP (NCBI Reference Sequence: NP_495713.2). Tree was reshaped on root with Newick Utilities v1.6 and visualized with Evolview v3.

The RdRp phylogenetic analysis revealed the presence of three main clades, each containing at least one protein sequence from *G. margarita* (Figure 2). A first clade includes, considering only the Mucoromycota, 2 *G. margarita* (g20441.t1, g4496.t1), 15 *R. irregularis*, and 2 *M. circinelloides* sequences, which are related to the *N. crassa* SAD-1. The second clade contains a single *G. margarita* (g5332.t1) and three *R. irregularis* sequences, which are related to *N. crassa* RRP-3, while the third one, including the homologous of *N. crassa* QDE-1, contains three *G. margarita* (g26014.t1, g25422.t1, and g15894.t1) and three *R. irregularis* sequences. The presence in *G. margarita* and *R. irregularis* of homologous sequences of the three well characterized *N. crassa* RdRp (Nakayashiki et al., 2006) suggests that AMF are equipped with a complete set of RdRp involved in the canonical *N. crassa* fungal RNAi pathway. Remarkably, one *G. margarita* RdRp (g15894.t1), which is distantly related to other fungal proteins, is characterized by the presence of some unusual “AAA” domains (“ATPases associated with diverse cellular activities”) at its C-terminal, which may suggest a distinct molecular function. Furthermore, we did not detect in *G. margarita* the occurrence of the small RdRp peptides found in *R. irregularis* (Silvestri et al., 2019).

Interestingly, we also found a protein of 232 amino acids (g12004.t1; not shown in the phylogenetic tree) containing an “RdRp_1” PFAM domain, which is a typical C-terminal domain of RdRp found in many eukaryotic viruses (Interpro ID: IPR001205). A blastp analysis against “non-redundant protein sequences” database on NCBI (*E*-value ≤ 1*e−*5) revealed that g12004.t1 is similar to a number of hypovirus- and fusarivirus sequences; the best viral hit is the polyprotein of *Cryphonectria* hypovirus 4 (NCBI accession: YP_138519.1). Notably, the same blast analysis highlighted five protein sequences of the phylogenetically related AMF *Gigaspora rosea* (NCBI accession: RIB25480, RIB25479, RIB01634, RIB24634, RIB12248). This result provides evidence of an endogenization event of a hypovirus presumably occurring in an ancestor of the *Gigaspora* lineage. It is worth noting that no hypovirus has ever been reported in AMF.

**FIGURE 3:**
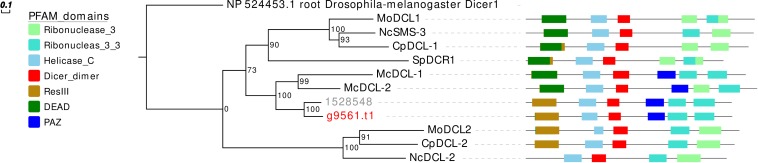
Phylogenetic analysis and PFAM domains of DCL proteins. Sequences are discernible by species according to a two-letter prefix and/or a color code: Mo, *Magnaporthe oryzae*; Nc, *Neurospora crassa*; Mc, *Mucor circinelloides*; Sp, *Schizosaccharomyces pombe*; Cp, *Cryphonectria parasitica*; gray, *Rhizophagus irregularis*; red, *Gigaspora margarita.* Protein ID (NCBI): MoMDL1 = XP_003714515.1, MoMDL2 = XP_003715365.1, NcSMS-3 = XP_961898.1, NcDCL-2 = XP_963538.3, SpDCR1 = NP_588215.2, McDCL-1 = CAK32533.1, McDCL-2 = CAZ65730.1, CpDCL-1 = ABB00356.1, CpDCL-2 = ABB00357.1. *R. irregularis* proteins are identified by JGI numeric codes. *G. margarita* proteins are identified by Venice et al. (2019) annotation code. The numbers at the nodes are bootstrap values (%) for 1000 replicates. Tree was rooted using *Drosophila melanogaster* Dicer 1 (NCBI Reference Sequence: NP_524453.1). Tree was reshaped on root with Newick Utilities v1.6 and visualized with Evolview v3.

Concerning the DCL phylogeny, the single *G. margarita* protein sequence clusters together with the *R. irregularis* one (Figure 3). However, the presence of one DCL may not be a common AMF trait, since we previously reported two sequences in *R. clarus* (Silvestri et al., 2019).

We then searched for evidence of expression of the genes encoding for the RNAi-related proteins exploiting the transcriptomic data published by Venice et al. (2019) obtained from four different conditions: germinating spores, strigolactone-treated spores, extraradical, and intraradical mycelium. All the genes are expressed in all the conditions (Supplementary Figure 1) with the only exception of the endogenized viral fragment (*g12004.t1*), which shows no expression or very low expression levels in symbiotic (extraradical and intraradical mycelium) and in the asymbiotic (spores) conditions, respectively.

In conclusion, the data confirmed that AMF are equipped with an RNAi machinery, characterized by the expansion of the *AGO-like* and, to some extent, the *RdRp* gene families. It would be interesting to understand whether these gene expansions were followed by functional differentiation, as happened to plant *AGO* (Poulsen et al., 2013).

It is tempting to speculate that this uncommonly high number of AGO-like could be related to the large amount of transposable elements of AMF genomes (Muszewska et al., 2017). In this context, we hypothesize that specific classes of AGO-like may be involved in the defense against mobile elements. It is worth noting that *G. margarita* lacks some genome defense mechanisms characterized in other fungi, such as fungal repeat-induced point (RIP) mutation and meiotic silencing of unpaired DNA (MSUD) (Venice et al., 2019); furthermore, based on AMF so far sequenced, TEs invasion seems to be specific of Gigasporaceae. In this context an efficient and fine-tuned anti-transposable elements defense system based on RNAi-related pathway could be instrumental in maintaining genome integrity. Moreover, since recent indirect evidences suggest that sRNAs are exchanged between plant and fungi in the AM symbiosis (Helber et al., 2011; Kikuchi et al., 2016; Tsuzuki et al., 2016; Xie et al., 2016; Voß et al., 2018), we speculate that the *AGO-like* gene expansion (and possibly their functional differentiation) in AMF mirrors the need to process, in a finely tuned way, the information that may come from the host plant. Further functional analyses will be needed to validate this hypothesis.

### *G. margarita* Is Characterized by a Peculiar Small RNA Population

**FIGURE 4:**
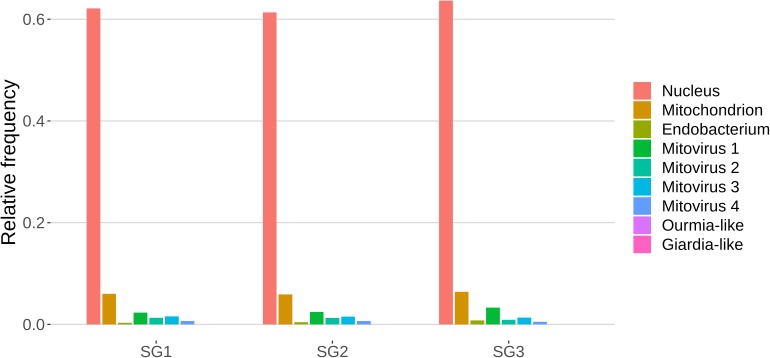
Relative mapping frequencies of small RNA to *Gigaspora margarita* metagenome (SG1, SG2, and SG3 refer to different libraries of “germinated spores” samples).

The role of sRNAs in AMF is still largely unknown. A preliminary characterization has been so far reported only for the model species *R. irregularis* (Silvestri et al., 2019). In this context the main focus of this work was to characterize the small RNAome of *G. margarita*, a species hosting a complex viral and endobacterial population (Ghignone et al., 2012; Turina et al., 2018) which has not been found in *R. irregularis* and *G. rosea* isolates so far. We sequenced three *G. margarita* sRNA libraries, each constructed with RNA extracted from germinated spores. The Illumina platform produced a total of 73,308,493 sRNA reads which were first cleaned up from adapters, artifacts, low-quality reads; after the removal of tRNA-, rRNA-, snRNA-, and snoRNA-related sequences, a total of 31,101,040 18–35-nt long reads were kept for further analysis (Supplementary Data Sheet 1). The resulting sRNA sequences were mapped with no mismatches to the nuclear and mitochondrial genomes of *G. margarita* (Pelin et al., 2012; Venice et al., 2019), to the genome of the endobacterium *Ca*Gg (Ghignone et al., 2012) and to the genomes of the six viruses (four mitoviruses, one Giardia-like, and one Ourmia-like virus) identified in *G. margarita* (Turina et al., 2018). Sixty-one to 64% of the reads, depending on the sRNA library, mapped exclusively to the nuclear genome, about 6% to the mitochondrial genome and the 0.3–0.8% to the endobacterial genome. The amount of reads uniquely mapping to the mitoviral genomes varied from 2.3 to 3.3% for Mitovirus 1 to 0.5–0.7% for Mitovirus 4. About 0.01% of the reads mapped to the genome of the Ourmia-like virus, while only about 0.0002% of total reads were associated with the Giardia-like virus (Figure 4). The reads mapped to both strands of viral genomes with different percentages (Supplementary Table 1). A limited number (0.13%) of total sRNA reads mapped to both nuclear genome and mitochondrial or endobacterial or mitoviral genomes (Supplementary Figure 2).

**FIGURE 5:**
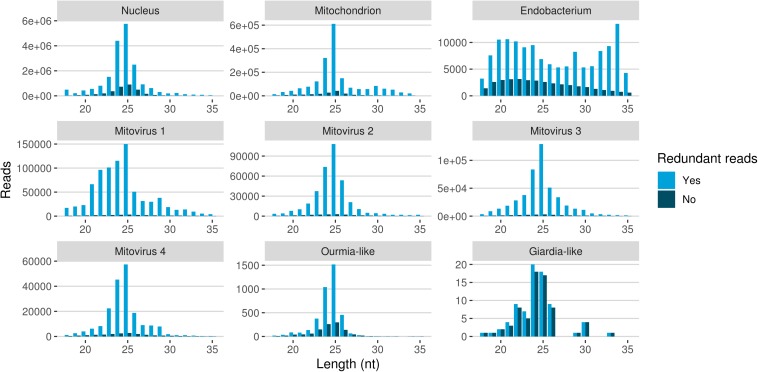
Nucleotide size distribution of sRNA reads (redundant and non-redundant) mapping to *G. margarita* metagenome.

The redundant and non-redundant sRNA reads mapping to all the different genomes, with the exception of the endobacterial one, were characterized by a similar unimodal nucleotide length distribution in which the most representative class consisted of the 25-nt long sequences (and 24-nt as the second most abundant class for all of them) (Figure 5). The accumulation of sRNAs with specific nucleotide lengths is commonly associated with the presence of an active sRNA-generating pathway (Mueth et al., 2015), since fungal species that do not possess a functional RNAi, such as *S. cerevisiae* (Drinnenberg et al., 2009), or DCL knock-out mutants (Raman et al., 2017) are not characterized by peaks over 20 nt. The nucleotide length distribution of the reads mapping to the endobacterial genome showed no evident peak; these sRNAs are likely to have originated by random degradation of longer transcripts, in accordance with the model that does not contemplate the presence of active RNAi mechanism in prokaryotes.

The nucleotide size distribution observed for *G. margarita* nuclear DNA mapping sRNAs (*Gma-*sRNAs; unimodal with maximum at 25-nt) is different from that of *R. irregularis* (bimodal with maxima at 24- or 26-nt and 31- to 33-nt; Silvestri et al., 2019). This result is not surprising considering that the length of the sRNAs seems not to be a conserved trait in fungi, even among species from the same genus, such as *Fusarium oxysporum* and *Fusarium graminearum* (Chen et al., 2014, 2015). For example *N. crassa* mainly produces 25-nt long sRNAs (Fulci and Macino, 2007), *M. circinelloides* 21- and 25-nt (Nicolás et al., 2003), *Aspergillus nidulans* 25-nt (Hammond and Keller, 2005), *M. oryzae* 19–23-nt (Kadotani et al., 2003), *Cryptococcus neoformans* 22-nt (Dumesic et al., 2013), *Trichoderma atroviride* 20–21- and 24-nt (Carreras-Villaseñor et al., 2013), *F. graminearum* 27- and 28-nt (Chen et al., 2015), *F. oxysporum* 19- and 21-nt (Chen et al., 2014), *Sclerotinia sclerotiorum* 22-nt (Derbyshire et al., 2019), and *Puccinia striiformis* 22-nt (Mueth et al., 2015).

The differences of the nuclear small RNAome composition between the two AMF can also be partially explained by the different experimental setups: while for *R. irregularis* we sequenced sRNAs from symbiotic conditions (fungal mycelium growing inside or outside plant roots), for *G. margarita* we sequenced sRNAs from asymbiotic condition (axenically germinated spores).

A further interesting observation was the high level of sRNA reads mapping to genomes of the four mitoviruses identified in *G. margarita* (Turina et al., 2018). Mitoviruses generally replicate in their host’s mitochondria (Hillman and Cai, 2013) thus in this case, exploiting the mitochondrial translation machinery, they rely on the mitochondrial translation code (such as the UGA codon for tryptophan, which in nuclear genetic code is a stop codon). Interestingly, *G. margarita* mitoviral genetic code lacks UGA codons and can be virtually translated in both cytosol and mitochondria (Turina et al., 2018). Recently, the sRNA response to a strictly mitochondrial plant mitovirus (Chenopodium quinoa mitovirus 1) was characterized: it has been shown that the mitovirus escaped the antiviral RNAi that normally originates 21–22 nt sRNA for cytoplasmic viruses. The overall number of sRNA of mitoviral origin was very low compared to a cytoplasmic virus, and the most represented length was 17 nt (Nerva et al., 2019), corresponding to the average size of sRNA originated inside plant mitochondria. In another study the sRNA response to a mitovirus in *F. circinatum* also pointed to a protection from the cytoplasmic antiviral RNAi since, also in this case, a relatively low accumulation of sRNA had the same length distribution of mitochondrial sRNAs (Muñoz-Adalia et al., 2018). A similar situation has been observed for the mitovirus infecting the ascomycete *C. parasitica* (Shahi et al., 2019). The analysis of mitoviral sRNA in *G. margarita* did not allow us to discern whether mitoviruses replicate in mitochondria or cytosol since, contrary to what happens in plants, we do not detect differences in the nucleotide length profiles for sRNAs with nuclear and mitochondrial origins. Both are in fact characterized by 25-nt long sRNA peaks, the same of sRNAs with mitoviral origin, suggesting the presence of similar sRNA-generating processes in the two cell compartments. Notably, this seems to be a specific feature of *G. margarita*; the analysis of nucleotide length profile of *R. irregularis* sRNAs (exploiting sRNA-seq data previously published; Silvestri et al., 2019) revealed that the population of mitochondrial DNA mapping sRNAs (decreasing curve from 18- to 35-nt) clearly differs from the population of nuclear DNA mapping sRNAs (Supplementary Figure 3).

The very high number of sRNA accumulating during mitovirus infection seem to suggest that, contrary to some of the systems described above, their RNA is indeed targeted by cytoplasmic RNAi, possibly during promiscuous replication (both mitochondrial and cytoplasmic). If this is a true antiviral response remains to be established. Another characteristic of *G. margarita* mitoviruses is the production during replication of episomic DNA fragments corresponding to their sequence (Turina et al., 2018): our sRNA analysis also aimed at searching for evidence of a specific anti-viral response originated by such DNA fragments, in analogy to the PIWI sRNA response originated by DNA fragments to control RNA viruses in insects (Goic et al., 2016). However, we could not detect any peculiar population of viral-derived sRNA (such as the insect PIWI sRNA) that allowed us to envisage a similar role for *G. margarita* viral DNA fragments.

**FIGURE 6:**
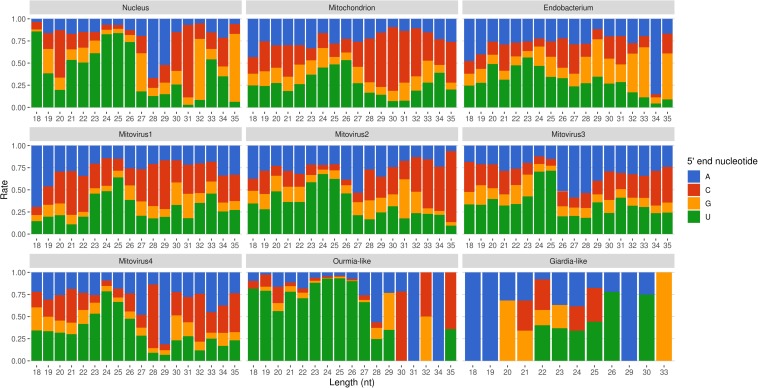
Relative nucleotide frequency of the 5′-ends of the sRNAs reads mapping to *Gigaspora margarita* metagenome (normalized on nucleotide composition of each genome).

Mapped sRNA reads were also analyzed for their 5′-end nucleotide composition (normalized on nucleotide composition of each genome). A general enrichment in uracil was observed for 23–26-nt long sequences with few differences depending on their genomic origin (Figure 6). Interestingly, the 23–26 nt range also corresponded to the group of the most expressed sRNAs (Figure 5), with the only exception of the endobacterium and the Giardia-like virus, the latter however characterized by very few total mapped reads. The 5′-ends enrichment in uracil, which is a rather common feature of fungal sRNA (Nicolás et al., 2003; Dumesic et al., 2013; Mueth et al., 2015; Nguyen et al., 2018; Derbyshire et al., 2019; Silvestri et al., 2019), for the 23–26-nt long *G. margarita* sRNAs (the most expressed ones), could be a further indication of a functional role. Remarkably, the nucleotide composition of the 5′-end of sRNAs affects their ability to be loaded onto different classes of AGO proteins; in *A. thaliana* the uracil at the 5′-end is indeed associated with the sRNA loading onto AGO1 and AGO10, while cytosine is associated with AGO5, and adenine with AGO2, AGO4, AGO6, AGO7, and AGO9 (Borges and Martienssen, 2015). Furthermore, it is worth noting that the 5′-end nucleotide compositions of the 23–26 nt range mitoviral sRNAs is enriched in uracil. This is a common feature observed for several mycoviruses (Donaire and Ayllón, 2017).

All these findings suggest the presence of active molecular pathways producing sRNAs, with different genomic origins (nuclear, mitochondrial, or viral). The sRNA peaks at 25 nt for all the reads mapping to different genomes (with the exception of the endobacterial one), together with the uracil enrichment at their 5′-end, suggests the presence of an RNAi pathway in *G. margarita* able to process sRNAs with nuclear and mitochondrial origin and also able to specifically target viral sequences.

### *G. margarita* Is Characterized by Different Populations of Nuclear sRNA-Generating Loci

Using ShortStack (Johnson et al., 2016) we performed a prediction and annotation of *G. margarita* nuclear genome loci producing sRNAs (*Gma*-sRNA-generating loci). Applying the same parameters used in a previous study (Silvestri et al., 2019), we predicted 4575 loci of which 3422 (75%) localized in intergenic regions and 1153 (25%) overlapped with predicted protein-encoded genes (i.e., loci that shared, for at least one nucleotide, the same genomic positions of elements reported as “mRNA” in the annotation file; Supplementary Data Sheet 2). A different situation was reported in *R. irregularis* that showed a predominance of sRNA-generating loci overlapping with protein-encoding genes (67% of the total; Silvestri et al., 2019). This could be due to a higher occurrence of intergenic regions in the genome of *G. margarita* considering that its very large genome contains a number of predicted genes similar to *R. irregularis* (Venice et al., 2019).

A total of 762 (17%) *Gma*-sRNA-generating loci showed similarity to fungal repetitive elements from RepBase 23.04 (Supplementary Data Sheet 2), in analogy to the 11% observed in *R. irregularis* (Silvestri et al., 2019). A further analysis of the genomic positions of the 4575 *Gma*-sRNA-generating loci revealed that 3635 (79%) overlapped for at least one nucleotide with *G. margarita* transposable elements (Supplementary Data Sheet 2); 672 of these loci overlapped on both transposable elements and protein-encoding genes.

**FIGURE 7:**
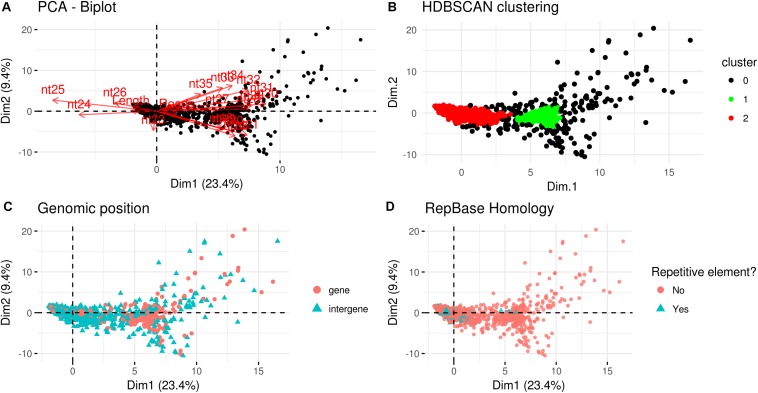
Characterization of *Gigaspora margarita(Gma)*-sRNA generating loci. **(A)** Biplot of principal components 1 and 2 of PCA based on the length of loci, the total number of mapped reads and the nucleotide size proportion of *Gma*-sRNAs (from 18 to 35 nt) defining each locus (19 total variables). **(B)** HDBSCAN clustering reveals the presence of two distinct populations of data (Clusters 1 and 2). **(C)** Overview of the relative genomic positions of the loci compared to those of protein-encoding genes. **(D)** Overview of the homology of *Gma-*sRNA-generating loci with fungal repetitive elements in RepBase 23.04 (tblastx: *E*-value ≤ 0.00005).

**FIGURE 8:**
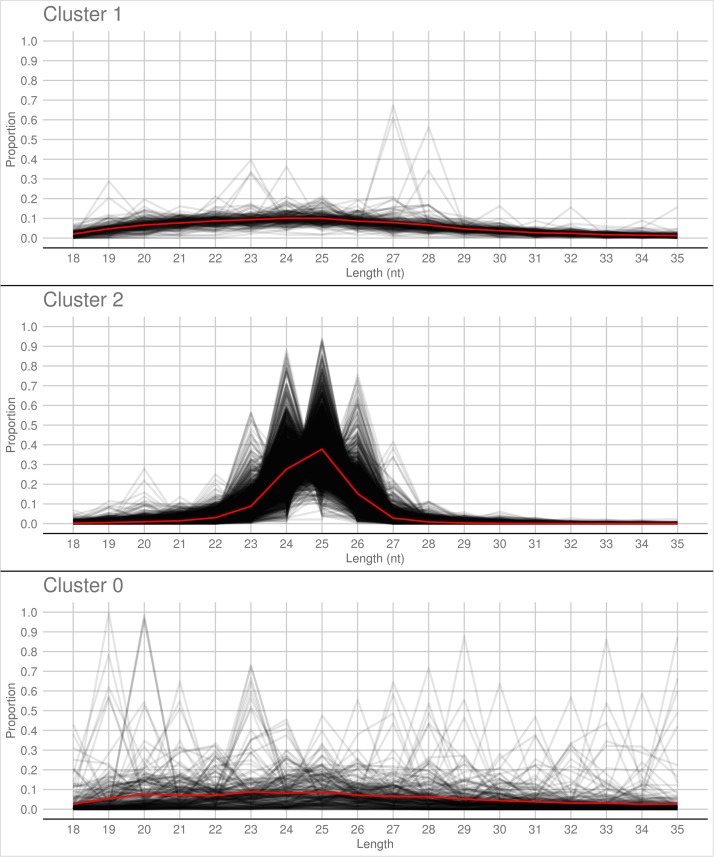
Nucleotide size distribution of sRNA reads that define the *Gigaspora margarita*-sRNA-generating loci of Cluster 1, Cluster 2, and Cluster 0 (the latter containing the non-clustered loci) according to HDBSCAN clustering. Black lines refer to the nucleotide size distribution of the sRNA reads defining the individual loci and red lines to the average nucleotide size distribution of each cluster.

A PCA analysis (Figure 7) on nuclear sRNA-generating loci was then performed, as previously proposed (Fahlgren et al., 2013), taking into account 19 independent variables per locus (locus length in nucleotide, total number of mapped reads, and their nucleotide size proportion from 18- to 35-nt). The 23.4 and 9.4% of the total variance were explained by PC1 (principal component 1) and PC2, respectively. Six variables (the proportion of 30-, 31-, 29-, 28-, and 25-nt long sequences) contribute for >50% to PC1 (Figure 7A), which mainly separates the loci in two groups, as confirmed by HDBSCAN (density-based spatial clustering of applications with noise) algorithm (Campello et al., 2015; Figure 7B). The two clusters, “cluster 1” and “cluster 2,” were composed by 172 and 4180 loci, respectively, while the remaining 223 loci were not clustered (we renamed this group as “cluster 0”; Supplementary Data Sheet 2). The nucleotide size distributions of the sRNA reads mapping to the loci belonging to the three clusters showed different average profiles. In particular, we observed an unimodal curve with the maximum peak at 25 nt for cluster 2, and a flat curve for cluster 0 and cluster 1, the latter however slightly enriched in 22–26 nt long sequences (Figure 8).

The loci belonging to cluster 2 could be further differentiated in different groups based on their maximum peaks. In fact, while 25 nt was the most represented size for the majority of them (3091 loci), the remaining were characterized by maximum peaks at 23 nt (31 loci), 24 nt (901 loci), 26 nt (113 loci), 27 nt (2 loci), or at both 24 and 25 nt (22 loci) (Supplementary Figure 4). The presence of loci producing sRNAs of different length suggests the existence of at least partially different sRNA-generating processes, specifically acting on each group of sRNA-generating loci from “cluster 2.” Unfortunately, the lack of stable genetic transformation protocols for AMF, and so the possibility to obtain DCL knock-out mutants, makes it difficult to understand whether all these non-25-nt-sRNA-generating loci are dependent, for sRNA production, on the single *G. margarita* DCL or whether other DCL-independent processes are involved, as reported in the basal fungus *M. circinelloides* (Torres-Martínez and Ruiz-Vázquez, 2016). The setup of complementation assays, in which *G. margarita* DCL is expressed in *M. circinelloides* DCL knock-out mutants, could help understanding whether the *G. margarita* single DCL participate in the biogenesis of the non-25-nt long sRNAs.

The loci belonging to different clusters also differentiated on the base of their relative genomic positions, with an amount of intergenic loci of about 62 and 78% for cluster 0 and cluster 2, respectively, as opposed to the 23% from cluster 1 (Figure 7C). A further differentiation was evident analyzing the percentage of repetitive elements homologous, with only 2 and 1% of the total loci from cluster 0 and cluster 1, opposed to the 18% from cluster 2 (Figure 7D).

The comparison with *R. irregularis* revealed that the sRNA-generating loci belonging to “Cluster 2” in both species share several features, such as the relative amount of intergenic loci (63 vs. 78%; *R. irregularis* vs. *G. margarita*) and repetitive elements homologs (16 vs. 18%) and the average length of mapped sRNAs (unimodal curve with maximum at 24-nt vs. unimodal curve with maximum at 25-nt). Instead, for “Cluster 1,” the differences between the two species are more relevant, relative to both percentage of total loci belonging to cluster (52 vs. 4%) and average length of mapped sRNAs (decreasing curve from 18- to 35-nt with no evident peaks vs. flat curve slightly enriched in 22- to 26-nt long sequences).

A blastn analysis revealed that 261 *Gma*-sRNA-generating loci are highly similar to 132 *R. irregularis*-sRNA-generating loci (*E*-value ≤ 1*e−*5); 135 out of 261 are intergenic loci (of which 18 correspond to transposable elements), while the remaining ones overlap with annotated genes (Supplementary Data Sheet 2). The presence of different populations of nuclear loci producing sRNAs may indicate the occurrence of different sRNA-generating processes.

**FIGURE 9:**
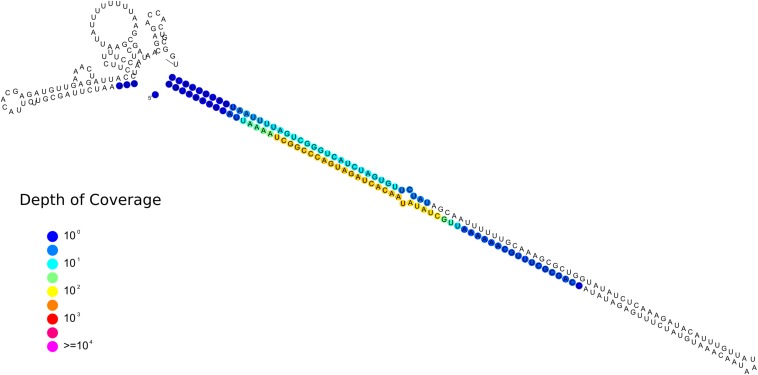
Predicted secondary structures of the putative *Gigaspora margarita* miRNA-like (Locus 1809; scaffold_534:156601-156872, positive strand) with color-coded sRNA-seq coverage per nucleotide.

One sRNA-generating locus (Locus 1809) was predicted as miRNA-like by ShortStack (Figure 9). Together with the 10 previously annotated in *R. irregularis*, this is the first evidence of the presence miRNA-like loci in basal fungi as no miRNA-like sequence was described in *M. circinelloides* (Torres-Martínez and Ruiz-Vázquez, 2016).

Finally, we observed that the genomic coordinates of the gene encoding for the endogenized viral RdRp fragment (*g12004.t1*; scaffold_323, bp from 157181 to 157959, positive strand) is totally contained inside the genomic boundaries of the sRNA-generating locus 1266 (scaffold_323; bp from 155733 to 160403) but on the opposite strand (since about the 98% of locus 1266 sRNAs mapped to the negative strand with a coverage of about 317 reads per million; Supplementary Data Sheet 2). Considering that the RNA-seq data from germinated spores pointed to a very low expression level for g12004.t1 (Supplementary Figure 1), we can hypothesize that the occurrence of abundant antisense sRNAs is evidence of a silencing activity toward the endogenized virus fragment.

### *G. margarita* Fungal/Viral sRNA Can Potentially Target Plant Transcripts

Cross-kingdom RNAi is nowadays recognized as a key mechanism involved in several plant–microbe interactions (Huang et al., 2019). Some experiments, which applied HIGS and VIGS techniques on mycorrhizal plants, suggested that sRNAs can also be exchanged between plants and AMF (Helber et al., 2011; Kikuchi et al., 2016; Tsuzuki et al., 2016; Voß et al., 2018), including *G. margarita* (Xie et al., 2016). In this context, we performed an *in silico* analysis in order to identify *G. margarita* sRNAs potentially able to silence plant transcripts, using *Medicago truncatula* as model organism (Bell, 2001). Our work is based on the assumption that some sRNAs from germinated spores are also maintained in the symbiotic phase; we focused therefore only on the most expressed (>100 RPM) 21-nt long *Gma*-sRNAs. The 21-nt long sRNAs are indeed generally involved in plants post-transcriptional gene silencing and, in addition, they are generally loaded onto AGO1 (Axtell, 2013), the only class of AGO known so far to be involved in cross-kingdom RNAi (Weiberg et al., 2013; Wang et al., 2016; Cui et al., 2019; Ren et al., 2019). Following this approach, we identified 292 *M. truncatula* mRNAs potentially targeted by 27 *Gma*-sRNAs (Supplementary Data Sheet 3), enriched in three GO terms: “signal transducer activity” (GO:0004871), “molecular transducer activity” (GO:0060089), and “lipid binding” (GO:0008289), according to Plant GO Slim ontology. A similar analysis, conducted on the most abundant (>100 RPM) 21-nt long sRNAs of the AMF *R. irregularis* (Rir-sRNAs; Silvestri et al., 2019), revealed 663 *M. truncatula* transcripts as putative targets of 57 *Rir*-sRNAs (Supplementary Data Sheet 4). Eleven plant mRNA sequences were predicted as targets of both *Gma-*sRNAs and *Rir*-sRNAs (Supplementary Table 2); notably, three of them – a “chitinase” (AES62408), an “expansin A10” (AES77475), and a “CCR4-NOT transcription complex protein” (AET01158) encoding genes – are targeted at the same site by sRNAs of both AMF (Supplementary Data Sheets 3, 4), reinforcing the hypothesis of a possible involvement of these sRNAs in host gene regulation in the AM symbiosis.

Since we assumed a possible high rate of false positives of these *in silico* prediction, as a negative control, we also performed a further target prediction on *M. truncatula* transcriptome using sRNAs from the unrelated non-mycorrhizal fungus *Aspergillus fumigatus* (Özkan et al., 2017). We identified 309 plant mRNA targets of 43 abundant (>100 RPM) *A. fumigatus* sRNAs (*Afu-*sRNAs; Supplementary Data Sheet 5). Among these, six plant mRNAs were predicted as targets of both *Gma-*sRNAs and *Afu-*sRNAs, six of both *Rir*-sRNAs and *Afu-*sRNAs and one (KEH44371, an “Ubiquitin-conjugating enzyme E2”) of sRNAs from all three fungal species (Supplementary Table 2). These results confirm that *in silico* target prediction analyses must only be intended as a preliminary step for the identification of target genes, especially in plant–microbe cross-kingdom interactions. *In vivo* experiments would be necessary to demonstrate the AMF sRNA silencing effect against *in silico* identified target *M. truncatula* sequences (Zanini et al., 2018). Our results also suggest that additional sRNA data from a higher number of AMF species will be instrumental to increase the robustness of *in silico* target prediction to identify conserved AMF sRNA effectors as well as plant targets.

We also investigated the potential involvement of *G. margarita* viral sRNAs in the regulation of plant mRNAs through cross-kingdom RNAi: we detected, by the *in silico* analysis, 248 plant transcripts potentially targeted by the 55 most expressed (>100 RPM) 21-nt long sRNAs derived from *G. margarita* mitoviruses (Supplementary Data Sheet 6). Although the role of viral-derived sRNAs in host plant gene regulation has been extensively studied and characterized (Shimura et al., 2011; Smith et al., 2011; Adkar-Purushothama et al., 2015; Yang et al., 2019), this is a first evidence of their potential implication in cross-kingdom RNAi from a fungus to a plant.

In conclusion, our work indicates that several AM fungal/viral sRNAs could potentially target plant transcripts and could act, if they are transferred into plant host cells, as RNA effectors in the AM symbiosis. Further experiments are needed to validate these putative fungal/viral sRNA – plant mRNA target pairs.

## Conclusion

Our work demonstrates that AMF *G. margarita* is equipped with a complete RNAi machinery and, in analogy to *Rhizophagus* species, shows a peculiar expansion of the *AGO* gene family. We also provided the first characterization of the small RNAome of a complex symbiotic meta-genome that involves a fungus permanently associated to bacteria and viruses. Furthermore, our results point to a possible cross-kingdom interaction with the plant host mediated by some of these sRNAs, including those of viral origin.

Mining additional AMF genomes and small RNAome will provide new insights on how these ancient microbes are able to establish long-lasting interactions with plants, bacteria, and viruses.

## Data Availability Statement

The sRNA-seq datasets analyzed in this study are available on European Nucleotide Archive (ENA) under the study accession number PRJEB35457.

## Author Contributions

LL, AS, and MT designed the experiments. AS, VF, LM, and FV performed the experiments and together with PB, MT, and LL carried out the data analyses. AS and LL wrote the manuscript.

## Conflict of Interest

The authors declare that the research was conducted in the absence of any commercial or financial relationships that could be construed as a potential conflict of interest.

## Footnotes

1 https://rpkgs.datanovia.com/factoextra/

2 https://www.girinst.org/repbase/

3 https://github.com/MikeAxtell/strucVis

## References

[B1] Adkar-Purushothama C. R. Brosseau C. Gigu E. Sano T. Moffett P. Perreaulta J. P. (2015). Small RNA derived from the virulence modulating region of the potato spindle tuber viroid silences callose synthase genes of tomato plants. *Plant Cell* 27 2178–2194. 10.1105/tpc.15.00523 26290537 PMC4568511

[B2] Axtell M. J. (2013). Classification and comparison of small RNAs from plants. *Annu. Rev. Plant Biol.* 64 137–159. 10.1146/annurev-arplant-050312-120043 23330790

[B3] Bell C. J. (2001). The Medicago Genome Initiative: a model legume database. *Nucleic Acids Res.* 29 114–117. 10.1093/nar/29.1.114 11125064 PMC29836

[B4] Borges F. Martienssen R. A. (2015). The expanding world of small RNAs in plants. *Nat. Publ. Gr.* 16 1–15. 10.1038/nrm4085 26530390 PMC4948178

[B5] Camacho C. Coulouris G. Avagyan V. Ma N. Papadopoulos J. Bealer K. (2009). BLAST+: architecture and applications. *BMC Bioinform.* 10:421. 10.1186/1471-2105-10-421 20003500 PMC2803857

[B6] Campello R. J. G. B. Moulavi D. Zimek A. Sander J. (2015). Hierarchical density estimates for data clustering, visualization, and outlier detection. *ACM Trans. Knowl. Discov. Data* 10 1–51. 10.1145/2733381

[B7] Carreras-Villaseñor N. Esquivel-Naranjo E. U. Villalobos-Escobedo J. M. Abreu-Goodger C. Herrera-Estrella A. (2013). The RNAi machinery regulates growth and development in the filamentous fungus *Trichoderma atroviride*. *Mol. Microbiol.* 89 96–112. 10.1111/mmi.12261 23672609

[B8] Chang S.-S. Zhang Z. Liu Y. (2012). RNA interference pathways in fungi: mechanisms and functions. *Annu. Rev. Microbiol.* 66 305–323. 10.1146/annurev-micro-092611-150138 22746336 PMC4617789

[B9] Chen E. C. H. Morin E. Beaudet D. Noel J. Yildirir G. Ndikumana S. (2018). High intraspecific genome diversity in the model arbuscular mycorrhizal symbiont *Rhizophagus irregularis*. *New Phytol.* 220 1161–1171. 10.1111/nph.14989 29355972

[B10] Chen R. Jiang N. Jiang Q. Sun X. Wang Y. Zhang H. (2014). Exploring microRNA-like small RNAs in the filamentous fungus *Fusarium oxysporum*. *PLoS One* 9:E104956. 10.1371/journal.pone.0104956 25141304 PMC4139310

[B11] Chen Y. Gao Q. Huang M. Liu Y. Liu Z. Liu X. (2015). Characterization of RNA silencing components in the plant pathogenic fungus *Fusarium graminearum*. *Sci. Rep.* 5:12500. 10.1038/srep12500 26212591 PMC4515635

[B12] Chow F. W. N. Koutsovoulos G. Ovando-Vázquez C. Neophytou K. Bermúdez-Barrientos J. R. Laetsch D. R. (2019). Secretion of an Argonaute protein by a parasitic nematode and the evolution of its siRNA guides. *Nucleic Acids Res.* 47 3594–3606. 10.1093/nar/gkz142 30820541 PMC6468290

[B13] Cui C. Wang Y. Liu J. Zhao J. Sun P. Wang S. (2019). A fungal pathogen deploys a small silencing RNA that attenuates mosquito immunity and facilitates infection. *Nat. Commun.* 10:4298. 10.1038/s41467-019-12323-12321 31541102 PMC6754459

[B14] Dai X. Zhuang Z. Zhao P. X. (2018). psRNATarget: a plant small RNA target analysis server (2017 release). *Nucleic Acids Res.* 46 W49–W54. 10.1093/nar/gky316 29718424 PMC6030838

[B15] Derbyshire M. Mbengue M. Barascud M. Navaud O. Raffaele S. (2019). Small RNAs from the plant pathogenic fungus *Sclerotinia sclerotiorum* highlight host candidate genes associated with quantitative disease resistance. *Mol. Plant Pathol.* 20 1279–1297. 10.1111/mpp.12841 31361080 PMC6715603

[B16] Donaire L. Ayllón M. A. (2017). Deep sequencing of mycovirus-derived small RNAs from *Botrytis* species. *Mol. Plant Pathol.* 18 1127–1137. 10.1111/mpp.12466 27578449 PMC6638239

[B17] Drinnenberg I. A. Weinberg D. E. Xie K. T. Mower J. P. Wolfe K. H. Fink G. R. (2009). RNAi in budding yeast. *Science* 326 544–550. 10.1126/science.1176945 19745116 PMC3786161

[B18] Dumesic P. A. Natarajan P. Chen C. Drinnenberg I. A. Schiller B. J. Thompson J. (2013). Stalled spliceosomes are a signal for RNAi-mediated genome defense. *Cell* 152 957–968. 10.1016/j.cell.2013.01.046 23415457 PMC3645481

[B19] Fahlgren N. Bollmann S. R. Kasschau K. D. Cuperus J. T. Press C. M. Sullivan C. M. (2013). *Phytophthora* have distinct endogenous small RNA populations yhat include short interfering and microRNAs. *PLoS One* 8:e77181. 10.1371/journal.pone.0077181 24204767 PMC3804510

[B20] Fulci V. Macino G. (2007). Quelling: post-transcriptional gene silencing guided by small RNAs in *Neurospora crassa*. *Curr. Opin. Microbiol.* 10 199–203. 10.1016/j.mib.2007.03.016 17395524

[B21] Ghignone S. Salvioli A. Anca I. Lumini E. Ortu G. Petiti L. (2012). The genome of the obligate endobacterium of an AM fungus reveals an interphylum network of nutritional interactions. *ISME J.* 6 136–145. 10.1038/ismej.2011.110 21866182 PMC3246228

[B22] Goic B. Stapleford K. A. Frangeul L. Doucet A. J. Gausson V. Blanc H. (2016). Virus-derived DNA drives mosquito vector tolerance to arboviral infection. *Nat. Commun.* 7:12410. 10.1038/ncomms12410 27580708 PMC5025746

[B23] Hahsler M. Piekenbrock M. Derek D. (2019). dbscan: fast density-based clustering with R. *J. Stat. Softw.* 91 1–30. 10.18637/jss.v091.i01

[B24] Hammond T. M. Keller N. P. (2005). RNA silencing in *Aspergillus nidulans* is independent of RNA-dependent RNA polymerases. *Genetics* 169 607–617. 10.1534/genetics.104.035964 15545645 PMC1449118

[B25] Helber N. Wippel K. Sauer N. Schaarschmidt S. Hause B. Requena N. (2011). A versatile monosaccharide transporter that operates in the arbuscular mycorrhizal fungus *Glomus* sp is crucial for the symbiotic relationship with plants. *Plant Cell* 23 3812–3823. 10.1105/tpc.111.089813 21972259 PMC3229151

[B26] Hillman B. I. Cai G. (2013). *The Family Narnaviridae. Simplest of RNA Viruses*, 1st Edn, Amsterdam: Elsevier Inc, 10.1016/B978-0-12-394315-6.00006-4 23498906

[B27] Hoang D. T. Chernomor O. von Haeseler A. Minh B. Q. Vinh L. S. (2018). UFBoot2: improving the ultrafast bootstrap approximation. *Mol. Biol. Evol.* 35 518–522. 10.1093/molbev/msx281 29077904 PMC5850222

[B28] Huang C.-Y. Wang H. Hu P. Hamby R. Jin H. (2019). Small RNAs – big players in plant-microbe interactions. *Cell Host Microb.* 26 173–182. 10.1016/j.chom.2019.07.021 31415750

[B29] Ipsaro J. J. Joshua-Tor L. (2015). From guide to target: molecular insights into eukaryotic RNA-interference machinery. *Nat. Struct. Mol. Biol.* 22 20–28. 10.1038/nsmb.2931 25565029 PMC4450863

[B30] Johnson N. R. Yeoh J. M. Coruh C. Axtell M. J. (2016). Improved placement of multi-mapping small RNAs. *G3* 6 2103–2111. 10.1534/g3.116.030452 27175019 PMC4938663

[B31] Junier T. Zdobnov E. M. (2010). The Newick utilities: high-throughput phylogenetic tree processing in the UNIX shell. *Bioinformatics* 26 1669–1670. 10.1093/bioinformatics/btq243 20472542 PMC2887050

[B32] Kadotani N. Nakayashiki H. Tosa Y. Mayama S. (2003). RNA silencing in the phytopathogenic fungus *Magnaporthe oryzae*. *Mol. Plant Microb. Interact.* 16 769–776. 10.1094/MPMI.2003.16.9.769 12971600

[B33] Kalyaanamoorthy S. Minh B. Q. Wong T. K. F. von Haeseler A. Jermiin L. S. (2017). ModelFinder: fast model selection for accurate phylogenetic estimates. *Nat. Methods* 14 587–591. 10.1038/nmeth.4285 28481363 PMC5453245

[B34] Katoh K. Standley D. M. (2013). MAFFT multiple sequence alignment software version 7: improvements in performance and usability. *Mol. Biol. Evol.* 30 772–780. 10.1093/molbev/mst010 23329690 PMC3603318

[B35] Kikuchi Y. Hijikata N. Ohtomo R. Handa Y. Kawaguchi M. Saito K. (2016). Aquaporin-mediated long-distance polyphosphate translocation directed towards the host in arbuscular mycorrhizal symbiosis: application of virus-induced gene silencing. *New Phytol.* 211 1202–1208. 10.1111/nph.14016 27136716

[B36] Krüger M. Krüger C. Walker C. Stockinger H. Schüßler A. (2012). Phylogenetic reference data for systematics and phylotaxonomy of arbuscular mycorrhizal fungi from phylum to species level. *New Phytol.* 193 970–984. 10.1111/j.1469-8137.2011.03962.x 22150759

[B37] Lanfranco L. Fiorilli V. Gutjahr C. (2018). Partner communication and role of nutrients in the arbuscular mycorrhizal symbiosis. *New Phytol.* 220 1031–1046. 10.1111/nph.15230 29806959

[B38] Langmead B. Trapnell C. Pop M. Salzberg S. (2009). 2C- Ultrafast and memory-efficient alignment of short DNA sequences to the human genome. *Genome Biol.* 10:R25. 10.1186/gb-2009-10-3-r25 19261174 PMC2690996

[B39] Lê S. Josse J. François H. (2008). FactoMineR: an R package for multivariate analysis. *J. Stat. Softw.* 25 1–18.

[B40] Lee S. J. Kong M. Harrison P. Hijri M. (2018). Conserved proteins of the RNA interference system in the arbuscularmycorrhizal fungus rhizoglomus irregulare provide new insight into the evolutionary history of glomeromycota. *Genome Biol. Evol.* 10 328–343. 10.1093/gbe/evy002 29329439 PMC5786227

[B41] Mayoral J. G. Hussain M. Albert Joubert D. Iturbe-Ormaetxe I. O’Neill S. L. Asgari S. (2014). Wolbachia small noncoding RNAs and their role in cross-kingdom communications. *Proc. Natl. Acad. Sci. U.S.A.* 111 18721–18726. 10.1073/pnas.1420131112 25512495 PMC4284532

[B42] Moazed D. (2009). Small RNAs in transcriptional gene silencing and genome defence. *Nature* 457 413–420. 10.1038/nature07756 19158787 PMC3246369

[B43] Mueth N. A. Ramachandran S. R. Hulbert S. H. (2015). Small RNAs from the wheat stripe rust fungus (*Puccinia striiformis* f.sp. tritici). *BMC Genomics* 16:718 10.1186/s12864-015-1895-1894 PMC4578785 26391470

[B44] Muñoz-Adalia E. J. Diez J. J. Fernández M. M. Hantula J. Vainio E. J. (2018). Characterization of small RNAs originating from mitoviruses infecting the conifer pathogen *Fusarium circinatum*. *Arch. Virol.* 163 1009–1018. 10.1007/s00705-018-3712-3712 29353424

[B45] Muszewska A. Steczkiewicz K. Stepniewska-Dziubinska M. Ginalski K. (2017). Cut-and-Paste transposons in fungi with diverse lifestyles. *Genome Biol. Evol.* 9 3463–3477. 10.1093/gbe/evx261 29228286 PMC5751038

[B46] Nakayashiki H. Kadotani N. Mayama S. (2006). Evolution and diversification of RNA silencing proteins in fungi. *J. Mol. Evol.* 63 127–135. 10.1007/s00239-005-0257-252 16786437

[B47] Nawrocki E. P. Burge S. W. Bateman A. Daub J. Eberhardt R. Y. Eddy S. R. (2015). Rfam 12.0: updates to the RNA families database. *Nucleic Acids Res.* 43 D130–D137. 10.1093/nar/gku1063 25392425 PMC4383904

[B48] Nerva L. Vigani G. Di Silvestre D. Ciuffo M. Forgia M. Chitarra W. (2019). Biological and molecular characterization of Chenopodium quinoa mitovirus 1 reveals a distinct sRNA response compared to cytoplasmic RNA viruses. *J. Virol.* 93 1–17. 10.1128/jvi.01998-1918 PMC6430534 30651361

[B49] Nguyen L. T. Schmidt H. A. Von Haeseler A. Minh B. Q. (2015). IQ-TREE: a fast and effective stochastic algorithm for estimating maximum-likelihood phylogenies. *Mol. Biol. Evol.* 32 268–274. 10.1093/molbev/msu300 25371430 PMC4271533

[B50] Nguyen Q. Iritani A. Ohkita S. Vu B. V. Yokoya K. Matsubara A. (2018). A fungal Argonaute interferes with RNA interference. *Nucleic Acids Res.* 46 2495–2508. 10.1093/nar/gkx1301 29309640 PMC5946944

[B51] Nicolás F. E. Torres-Martínez S. Ruiz-Vázquez R. M. (2003). Two classes of small antisense RNAs in fungal RNA silencing triggered by non-integrative transgenes. *EMBO J.* 22 3983–3991. 10.1093/emboj/cdg384 12881432 PMC169057

[B52] Özkan S. Mohorianu I. Xu P. Dalmay T. Coutts R. H. A. (2017). Profile and functional analysis of small RNAs derived from *Aspergillus fumigatus* infected with double-stranded RNA mycoviruses. *BMC Genomics* 18:3778. 10.1186/s12864-017-3773-3778 28558690 PMC5450132

[B53] Pelin A. Pombert J. F. Salvioli A. Bonen L. Bonfante P. Corradi N. (2012). The mitochondrial genome of the arbuscular mycorrhizal fungus *Gigaspora margarita* reveals two unsuspected trans-splicing events of group I introns. *New Phytol.* 194 836–845. 10.1111/j.1469-8137.2012.04072.x 22320438

[B54] Poulsen C. Vaucheret H. Brodersen P. (2013). Lessons on RNA silencing mechanisms in plants from eukaryotic argonaute structures. *Plant Cell* 25 22–37. 10.1105/tpc.112.105643 23303917 PMC3584537

[B55] Quinlan A. R. Hall I. M. (2010). BEDTools: A flexible suite of utilities for comparing genomic features. *Bioinformatics* 26 841–842. 10.1093/bioinformatics/btq033 20110278 PMC2832824

[B56] Raman V. Simon S. A. Demirci F. Nakano M. Meyers B. C. Donofrio N. M. (2017). Small RNA functions are required for growth and development of *Magnaporthe oryzae*. *Mol. Plant Microb. Interact.* 30 517–530. 10.1094/MPMI-11-16-0236-R 28504560

[B57] Ren B. Wang X. Duan J. Ma J. (2019). Rhizobial tRNA-derived small RNAs are signal molecules regulating plant nodulation. *Science* 365 919–922. 10.1126/science.aav8907 31346137

[B58] Shahi S. Eusebio-Cope A. Kondo H. Hillman B. I. Suzuki N. (2019). Investigation of host range of and host defense against a mitochondrially replicating mitovirus. *J. Virol.* 93 1–15. 10.1128/jvi.01503-1518 30626664 PMC6401429

[B59] Shahid S. Kim G. Johnson N. R. Wafula E. Wang F. Coruh C. (2018). MicroRNAs from the parasitic plant *Cuscuta campestris* target host messenger RNAs. *Nature* 553 82–85. 10.1038/nature25027 29300014

[B60] Shimura H. Pantaleo V. Ishihara T. Myojo N. Inaba J. Sueda K. (2011). A viral satellite RNA induces yellow symptoms on tobacco by targeting a gene involved in chlorophyll biosynthesis using the RNA silencing machinery. *PLoS Pathog.* 7:e02021. 10.1371/journal.ppat.1002021 21573143 PMC3088725

[B61] Silvestri A. Fiorilli V. Miozzi L. Accotto G. P. Turina M. Lanfranco L. (2019). In silico analysis of fungal small RNA accumulation reveals putative plant mRNA targets in the symbiosis between an arbuscular mycorrhizal fungus and its host plant. *BMC Genomics* 20:169. 10.1186/s12864-019-5561-0 30832582 PMC6399891

[B62] Smith N. A. Eamens A. L. Wang M. B. (2011). Viral small interfering RNAs target host genes to mediate disease symptoms in plants. *PLoS Pathog.* 7:e02022. 10.1371/journal.ppat.1002022 21573142 PMC3088724

[B63] Spatafora J. W. Chang Y. Benny G. L. Lazarus K. Smith M. E. Berbee M. L. (2016). A phylum-level phylogenetic classification of zygomycete fungi based on genome-scale data. *Mycologia* 108 1028–1046. 10.3852/16-042 27738200 PMC6078412

[B64] Subramanian B. Gao S. Lercher M. J. Hu S. Chen W.-H. (2019). Evolview v3: a webserver for visualization, annotation, and management of phylogenetic trees. *Nucleic Acids Res.* 47 W270–W275. 10.1093/nar/gkz357 31114888 PMC6602473

[B65] Tian T. Liu Y. Yan H. You Q. Yi X. Du Z. (2017). AgriGO v2.0: a GO analysis toolkit for the agricultural community, 2017 update. *Nucleic Acids Res.* 45 W122–W129. 10.1093/nar/gkx382 28472432 PMC5793732

[B66] Torres-Martínez S. Ruiz-Vázquez R. M. (2016). RNAi pathways in Mucor: a tale of proteins, small RNAs and functional diversity. *Fungal Genet. Biol.* 90 44–52. 10.1016/j.fgb.2015.11.006 26593631

[B67] Tsuzuki S. Handa Y. Takeda N. Kawaguchi M. (2016). Strigolactone-induced putative secreted protein 1 is required for the establishment of symbiosis by the arbuscular mycorrhizal fungus *Rhizophagus irregularis*. *Mol. Plant Microb. Interact.* 29 1–59. 10.1094/MPMI-10-15-0234-R 26757243

[B68] Turina M. Ghignone S. Astolfi N. Silvestri A. Bonfante P. Lanfranco L. (2018). The virome of the arbuscular mycorrhizal fungus *Gigaspora margarita* reveals the first report of DNA fragments corresponding to replicating non-retroviral RNA viruses in Fungi. *Environ. Microbiol.* 20 2012–2025. 10.1111/1462-2920.14060 29393558

[B69] Venice F. Ghignone S. Salvioli A. Amselem J. Novero M. Xianan X. (2019). At the nexus of three kingdoms-: the genome of the mycorrhizal fungus *Gigaspora margarita* provides insights into plant, endobacterial and fungal interactions. *Environ Microbiol.* 22 122–141. 10.1111/1462-2920.14827 31621176

[B70] Voß S. Betz R. Heidt S. Corradi N. Requena N. (2018). RiCRN1, a crinkler effector from the arbuscular mycorrhizal fungus rhizophagus irregularis, functions in arbuscule development. *Front. Microbiol.* 9:2068. 10.3389/fmicb.2018.02068 30233541 PMC6131194

[B71] Wang M. Weiberg A. Lin F.-M. Thomma B. P. H. J. Huang H.-D. Jin H. (2016). Bidirectional cross-kingdom RNAi and fungal uptake of external RNAs confer plant protection. *Nat. Plants* 2:16151. 10.1038/nplants.2016.151 27643635 PMC5040644

[B72] Weiberg A. Wang M. Lin F. M. Zhao H. Zhang Z. Kaloshian I. (2013). Fungal small RNAs suppress plant immunity by hijacking host RNA interference pathways. *Science* 342 118–123. 10.1126/science.1239705 24092744 PMC4096153

[B73] Wilson R. C. Doudna J. A. (2013). Molecular mechanisms of RNA interference. *Annu. Rev. Biophys.* 42 217–239. 10.1146/annurev-biophys-083012-130404 23654304 PMC5895182

[B74] Xie X. Lin H. Peng X. Xu C. Sun Z. Jiang K. (2016). Arbuscular mycorrhizal symbiosis requires a phosphate transceptor in the *Gigaspora margarita* fungal symbiont. *Mol. Plant.* 9 1583–1608. 10.1016/j.molp.2016.08.011 27688206

[B75] Yang Y. Liu T. Shen D. Wang J. Ling X. Hu Z. (2019). Tomato yellow leaf curl virus intergenic siRNAs target a host long noncoding RNA to modulate disease symptoms. *PLoS Pathog.* 15:e07534. 10.1371/journal.ppat.1007534 30668603 PMC6366713

[B76] Zanini S. Šečić E. Jelonek L. Kogel K.-H. (2018). A bioinformatics pipeline for the analysis and target prediction of RNA effectors in bidirectional communication during plant-microbe interactions. *Front. Plant Sci.* 9:1212. 10.3389/fpls.2018.01212 30177942 PMC6109766

[B77] Zerbino D. R. Achuthan P. Akanni W. Amode M. R. Barrell D. Bhai J. (2018). Ensembl 2018. *Nucleic Acids Res.* 46 D754–D761. 10.1093/nar/gkx1098 29155950 PMC5753206

[B78] Zhang T. Zhao Y. L. Zhao J. H. Wang S. Jin Y. Chen Z. Q. (2016). Cotton plants export microRNAs to inhibit virulence gene expression in a fungal pathogen. *Nat. Plants* 2:16153. 10.1038/nplants.2016.153 27668926

