# Supplementary material for: Different Genetic Sources Contribute to the Small RNA Population in the Arbuscular Mycorrhizal Fungus Gigaspora margarita
Source: Front Microbiol. 2020 Mar 13;11:395. doi: 10.3389/fmicb.2020.00395 (PMC7082362; doi:10.3389/fmicb.2020.00395)
Supplement: Supplementary file 1 [file Table_1.pdf]

Supplementary Table 1. Number of sRNA reads (and relative percentage) mapping on specific strands of viral genomes

Genome strand	Mitovirus 1	Mitovirus 2	Mitovirus 3	Mitovirus 4	Ourmia-like	Giardia-like
Positive	576632 (71.7%)	174844 (47.2%)	209644 (44.9%)	55060 (27.8%)	755 (19.6%)	60 (77.9%)
Negative	228066 (28.3%)	195398 (52.8%)	257390 (55.1%)	142720 (72.2%)	3106 (80.4%)	17 (22.1%)
