# Supplementary material for: Different Genetic Sources Contribute to the Small RNA Population in the Arbuscular Mycorrhizal Fungus Gigaspora margarita
Source: Front Microbiol. 2020 Mar 13;11:395. doi: 10.3389/fmicb.2020.00395 (PMC7082362; doi:10.3389/fmicb.2020.00395)
Supplement: Supplementary file 2 [file Table_2.pdf]

Supplementary Table 2. List of *M. truncatula* transcripts identified as potential targets of fungal sRNAs of more than one species *(Gma = Gigaspora margarita, Rhi = Rhizophagus irregularis, Afu = Aspergillus fumigatus).*

Species	*M. truncatuala* target (Ensembl trasncript ID)	Target description
Gma + Rir	AES62408	Chitinase
Gma + Rir	AES71533	CASP ARALYDRAFT-like protein
Gma + Rir	AES77475	Expansin A10
Gma + Rir	AET01158	CCR4-NOT transcription complex protein; putative
Gma + Rir	KEH22883	Kinase 1B
Gma + Rir	KEH26114	plant/T31B5-30 protein
Gma + Rir	KEH27632	Auxin efflux carrier family protein
Gma + Rir	KEH28968	Hypothetical protein
Gma + Rir	KEH40512	armadillo/beta-catenin-like repeat protein
Gma + Rir	KEH41047	Dentin sialophosphoprotein; putative
Gma + Rir+ Afu	KEH44371	Ubiquitin-conjugating enzyme E2
Gma + Afu	AES94317	Malectin/receptor-like kinase family protein
Gma + Afu	KEH37565	DENN (AEX-3) domain protein
Gma + Afu	AES63168	MACPF domain protein
Gma + Afu	AES75598	leucine-rich receptor-like kinase family protein
Gma + Afu	AES98293	E3 ubiquitin-protein ligase UPL1-like protein
Rir + Afu	KEH36415	Ion channel pollux-like protein
Rir + Afu	AES63182	Ion channel pollux-like protein
Rir + Afu	AES88097	homeobox leucine zipper protein
Rir + Afu	AES67973	peptide/nitrate transporter plant
Rir + Afu	AES99248	senescence-associated protein, putative
