# Supplementary material for: Different Genetic Sources Contribute to the Small RNA Population in the Arbuscular Mycorrhizal Fungus Gigaspora margarita
Source: Front Microbiol. 2020 Mar 13;11:395. doi: 10.3389/fmicb.2020.00395 (PMC7082362; doi:10.3389/fmicb.2020.00395)
Supplement: Supplementary file 3 [file Image_1.pdf]

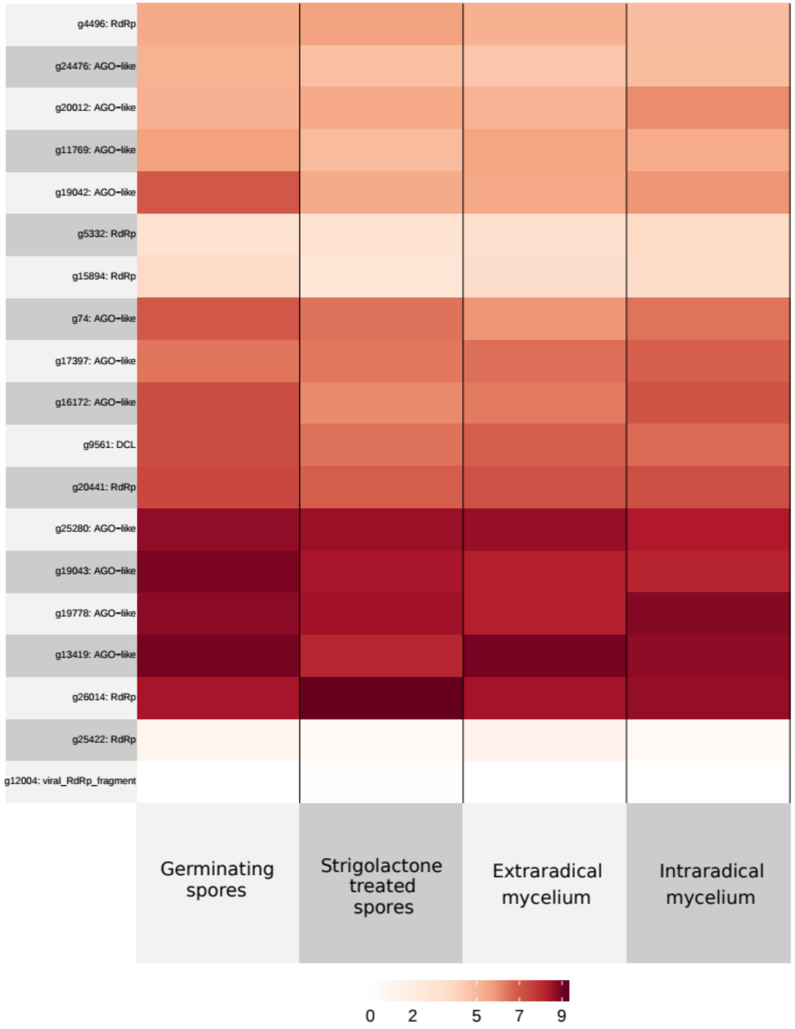

Supplementary Figure 1. Heatmap of expression levels (log2(mean TMM)) of *Gigaspora margarita* RNAi- related genes according to RNA-seq data analyzed by Venice et al. (2019)
