# Supplementary material for: Different Genetic Sources Contribute to the Small RNA Population in the Arbuscular Mycorrhizal Fungus Gigaspora margarita
Source: Front Microbiol. 2020 Mar 13;11:395. doi: 10.3389/fmicb.2020.00395 (PMC7082362; doi:10.3389/fmicb.2020.00395)
Supplement: Supplementary file 4 [file Image_2.pdf]

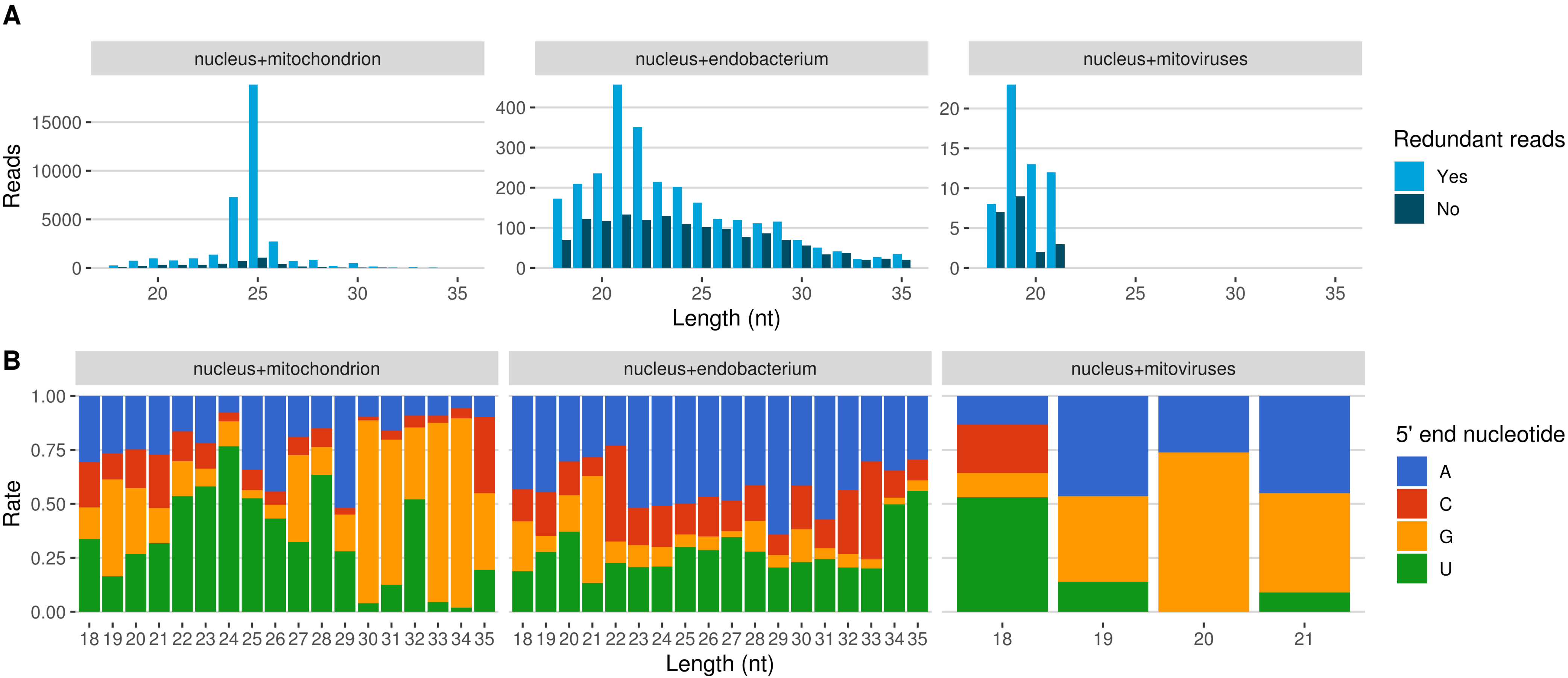

Supplementary Figure 2. Features of the sRNA reads mapping to more than one genome of *Gigaspora margarita* metagenome ("nucleus+mitochondrion", "nucleus+endobacterium" and "nucleus+mitoviruses" indicate which are the genomes). **(A)** Nucleotide size distribution (redundant and non-redundant) of the sRNA reads. **(B)** Relative nucleotide frequency of the 5'-ends of the sRNA reads.
