# Supplementary material for: Different Genetic Sources Contribute to the Small RNA Population in the Arbuscular Mycorrhizal Fungus Gigaspora margarita
Source: Front Microbiol. 2020 Mar 13;11:395. doi: 10.3389/fmicb.2020.00395 (PMC7082362; doi:10.3389/fmicb.2020.00395)
Supplement: Supplementary file 5 [file Image_3.pdf]

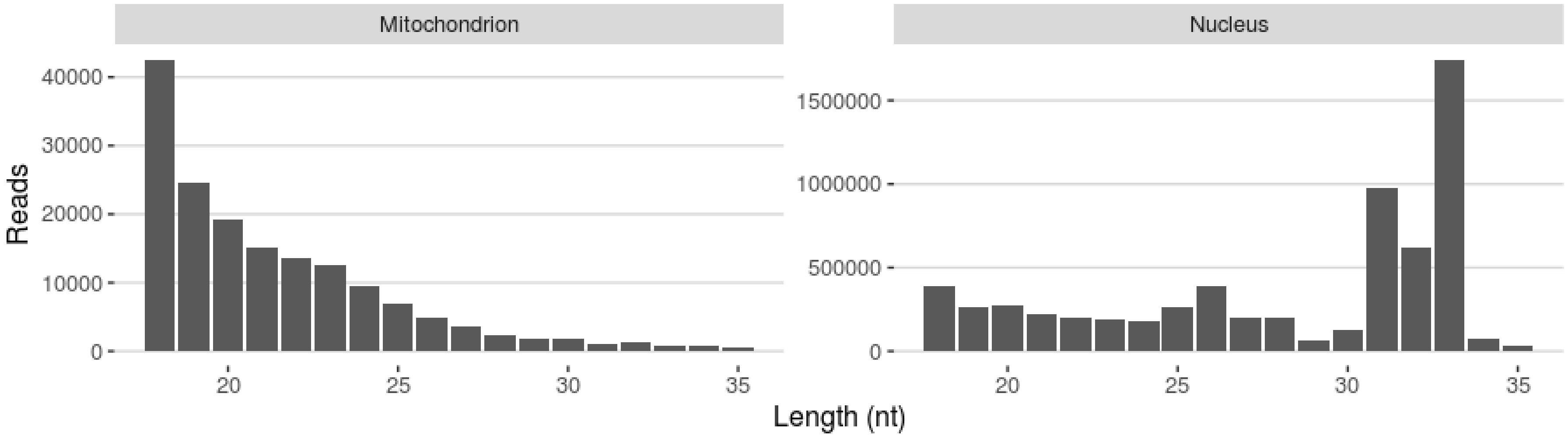

Supplementary Figure 3. Nucleotide size distribution of redundant sRNA reads from *Rhizophagus irregularis* extraradical mycelim samples (Silvestri et. 2019) mapping on *R. irregularis* mitochondrial (left) and nuclear genome (right).
