# Supplementary material for: Different Genetic Sources Contribute to the Small RNA Population in the Arbuscular Mycorrhizal Fungus Gigaspora margarita
Source: Front Microbiol. 2020 Mar 13;11:395. doi: 10.3389/fmicb.2020.00395 (PMC7082362; doi:10.3389/fmicb.2020.00395)
Supplement: Supplementary file 6 [file Image_4.pdf]

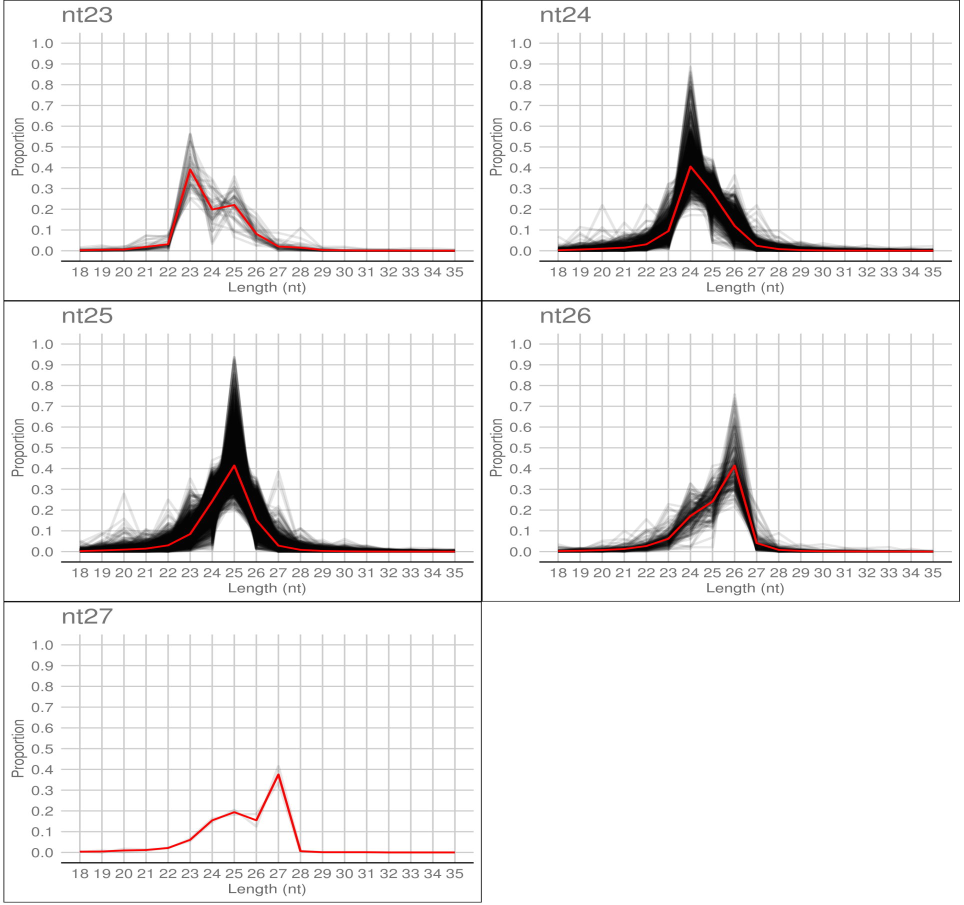

Supplementary Figure 4. Nucleotide size distribution of sRNA reads that define the *Gma*-sRNA-generating loci of Cluster 2, separated in groups based on their maximum peak. Black lines refer to the nucleotide size distribution of the sRNA reads defining the individual loci and red lines to the average nucleotide size distribution of each group.
